# Editorial: Current MEG Research in Psychiatry

**DOI:** 10.3389/fpsyt.2021.647085

**Published:** 2021-02-02

**Authors:** Yoji Hirano, Peter J. Uhlhaas

**Affiliations:** ^1^Department of Neuropsychiatry, Graduate School of Medical Sciences, Kyushu University, Fukuoka, Japan; ^2^Department of Psychiatry, Harvard Medical School, VA Boston Healthcare System, Boston, MA, United States; ^3^Department of Child and Adolescent Psychiatry, Charité - Universitätsmedizin, Berlin, Germany; ^4^Institute of Neuroscience and Psychology, University of Glasgow, Glasgow, United Kingdom

**Keywords:** magnetoencephalography, MEG, brain imaging, spatiotemporal resolution, neurobiology, psychiatric disorders, biomarkers

The identification of biomarkers for psychiatric disorders has the potential to revolutionize and redefine psychiatry, as these could aid diagnosis and identify novel treatment targets (1). Toward this goal, neuroimaging studies of psychiatric disorders have greatly accelerated over the past two decades. Specifically, neurophysiological approaches, such as electro- and magneto-encephalography (EEG/MEG) have revealed novel insights into sensory and cognitive abnormalities in psychiatric conditions due to their excellent temporal resolution in the ms range (2–10).

More than 50 years ago since the first MEG measurement of brain signals by Cohen (11), MEG is now a widely adopted method for the understanding of normal brain functions and their relationship to sensory and cognitive processes (12). MEG has an advantage of capturing neuronal dynamics with greater spatiotemporal and spectral detail than EEG. In addition, MEG has seen over the last years an emerging application as a tool to investigate pathophysiological mechanisms and biomarkers in psychiatric conditions (9). Given its exquisite temporal and good spatial resolution, MEG has provided novel insights into the role of rhythmic activity in autism spectrum disorder (ASD) and schizophrenia (SZ) (8, 9) as well as the first indication that MEG may contribute toward early detection and diagnosis in early-stage SZ and Alzheimer's disease (AD) (13, 14).

This special issue on “Current MEG Research in Psychiatry” provides a state-of-the-art overview of the potential and scope of MEG, ranging from recent developments in MEG methods (Hironaga et al.) to the study of cognitive and sensory dysfunctions in ASD (Yuk et al.) and SZ (Coffmann et al., Sauer et al., and Ohara et al.) including functional connectivity in psychosis (Candelaria-Cook and Stephen and Sunaga et al.), to the role of alterations nervous system (ANS) activity in major depression disorder (MDD) (Zhou et al.). While MEG has been largely used to study magnetic fields generated by the activity of the neural networks in the brain, cardio-vascular activity is also associated with a strong electrical dipole resulting in a measurable magnetic field. Two papers (Zhou et al., Kato et al.) use this approach to examine the relationship between ANS during emotion processing. Specifically, Zhou et al. show that the changes in heart rate variability (HRV) are associated with inhibitory dysfunctions of functional connectivity in the prefrontal cortex (PFC) in MDD while Kato et al. demonstrate changes in heart-rate evoked fields (HEFs) and disgust.

A particular focus of MEG-applications has been investigations into the neurophysiology of sensory and cognitive deficits in SZ and the papers by Coffmann et al., Sauer et al., and Ohara et al. provide novel perspectives on this topic. Coffmann et al. examined contralateral alpha suppression (CAS) during visual short-term memory and observed a failure to modulate alpha-band power in a load-dependent manner in first-episode SZ (FESZ) patients. Changes in neural oscillations and event-related fields (ERFs) during repetition suppression were investigated by Sauer et al. Analysis of virtual-channel MEG-data revealed that both repetition suppression and enhancement were impaired in a sample of chronic SZ patients as indicated by deficits in the modulation of beta/gamma-band power as well as in ERFs in thalamic and occipital cortices. Finally, Ohara et al. examined whether the M170 indexes a specific deficit in face-processing in SZ. Patients with SZ showed a selective deficit to face-stimuli in the fusiform face area (FFA) that correlated with the severity of negative symptoms.

Spectral signatures of resting-state activity are currently attracting a lot of attention in psychosis research (15–19) and the reports by Candelaria-Cook and Stephen as well as Sunaga et al. provide advanced perspectives on this theme. Candelaria-Cook and Stephen tested the reliability of MEG-based functional connectivity metrics in HC and SZ. They found that HC had higher reliability compared to SZ, and the default mode, cognitive control, and visual networks had higher test-retest reliability compared to somatosensory and auditory networks. In addition, both eyes open and eyes closed resting-state networks were found to be reliable over sessions. Sunaga et al. explored the frequency-specific resting-state connectome including limbic network (LM) and default mode network (DMN) in bipolar psychosis (BP) *via* a novel graph analysis. BP patients show frequency-specific alterations in the inter-community (between right LM–right DMN) in the gamma-band and intra-community edges (within left LM) in the high beta-band, and the intra-community edges in the left LM at beta-band frequencies were positively correlated with depressive symptoms.

Investigations into the developmental trajectories of potential biomarkers are also crucial for early detection and intervention, in particular for disorders with an onset in childhood, such as ASDs (20). A longitudinal MEG study by Edgar et al. examined the development of auditory cortex M50 and M100 ERFs in children (6–8 years of age). The authors show significant between-subject as well as within-subject (left- and right-hemisphere) variability, in particular for M100 responses during normal brain development. Yuk et al. examined phase-synchronization during working memory in adult with ASDs to investigate the contribution of long-range synchronization toward cognitive deficits. While adults with ASD appropriately employed alpha-band oscillations to facilitate maintenance of novel visual stimuli in short-term and working memory, the topology and networks involved were different from controls, indicating more effortful processing in adults with ASDs.

Hironaga et al. provide a state-of-the-art review on the future prospects of MEG research in psychiatric disorders through addressing key empirical and methodological challenges. Despite the relatively small number of systems worldwide and high maintenance costs (12), MEG has the potential to significantly contribute toward the development of biomarkers for early detection and diagnosis for major syndromes [e.g., (13)]. Groundbreaking innovations, such as the new optically-pumped magnetometer (OPM) or wearable OPMs (21), will lead to a deeper understanding of the neural basis of psychiatric disorders as well as facilitate the development of novel treatment targets including neurofeedback treatments (22). Accordingly, we believe that the continued development of MEG hardware and analyses approaches will open up new perspectives for establishing neurophysiological biomarkers for psychiatric disorders.

## Author Contributions

PU and YH prepared the first draft of the manuscript and edited the manuscript. All authors contributed to and have approved the final manuscript.

## Conflict of Interest

PU reports having received research funding from Lilly UK and Lundbeck UK outside the submitted work. The remaining author declares that the research was conducted in the absence of any commercial or financial relationships that could be construed as a potential conflict of interest.
